# Zinc transporters are differentially expressed in human non-small cell lung cancer

**DOI:** 10.18632/oncotarget.11884

**Published:** 2016-09-07

**Authors:** Cuiping Huang, Xiaobo Cui, Xiaotian Sun, Jingxuan Yang, Min Li

**Affiliations:** ^1^ Department of Internal Medicine, College of Clinical Medicine, Hubei University of Science and Technology, Xianning, Hubei 437100, China; ^2^ The Vivian L. Smith Department of Neurosurgery, the University of Texas Medical School at Houston, Houston, TX 77030, USA; ^3^ Department of Medicine, The University of Oklahoma Health Sciences Center, Oklahoma City, OK 73104, USA; ^4^ Department of Surgery, The University of Oklahoma Health Sciences Center, Oklahoma City, OK 73104, USA; ^5^ Harbin Medical University, Harbin, Heilongjiang 150081, China; ^6^ Department of Internal Medicine, Clinic of August First Film Studio, Beijing 100161, China

**Keywords:** zinc transporter, ZIP4, expression profiling, lung cancer

## Abstract

Lung cancer is one of the most common human malignancies worldwide, but its oncogenesis process remains unclear. Recent studies demonstrated that zinc (Zn) and Zn transporters were associated with the development and progression of human cancers. The role of Zn transporters including ZIPs and ZnTs in lung cancer, however, has never been evaluated. Thus, we aimed to investigate the expression levels of all human Zn transporters, including 14 ZIPs and 10 ZnTs, in eight different lung cancer cell lines and paired human tumor tissues. We observed great variations in ZIPs and ZnTs mRNA levels across cell lines and human lung cancer specimens. ZIPs showed a tendency to be upregulated, while ZnTs exhibited a downward expression trend. ZIP4 was overexpressed in six lung cancer cell lines and 59% (26/44) of tumor tissues, which was consistent with results from lung cancer datasets including TCGA database. Our results indicated that the dysregulation of Zn transporters may contribute to lung tumorigenesis.

## INTRODUCTION

Lung cancer is the most frequently occurring cancer worldwide and the leading cause of cancer-related death in the US, representing almost 14% of all cancers diagnosed and approximately 28% of all cancer deaths [1–4]. With a lack of reliable diagnostic markers at early stages of disease and ineffective therapies, the prognosis for lung cancer patients is poor. The 5-year overall survival for lung cancer at all stages is 16.3%; this rate is much lower than that of many other cancers, such as colon (65.2%), breast (90.0%), and prostate (99.9%) cancers, the survival rates of which have remained largely unchanged over the last two decades [5]. Despite the extensive research into diagnostic and therapeutic methods for this devastating disease, little is known of the pathogenesis and molecular mechanism of lung cancer. Therefore, identifying new markers and therapeutic targets for the purposes of interrupting infiltrative growth and developing novel treatments for lung cancer is needed.

While many etiological factors participate in the pathogenesis of lung cancer, increasing attention has been paid to the role of trace elements, especially zinc (Zn). Zn is an essential micronutrient, a metal belonging to the group IIb series, which is associated with the function of over 300 enzymes, thousands of proteins and transcription factors, and upwards of 10% of the human genome [6–9]. Zn plays a pivotal role in a series of basic biological processes, such as the metabolism of nucleic acids, proteins, carbohydrates and lipids, and the regulation of gene transcription, cell proliferation, and differentiation [10]. Zn deficiency could impair DNA synthesis, decrease food intake, and induce growth retardation, immune damage, and severe dermatitis [11, 12]. However, excessive Zn may also exert cytotoxic effects [13].

The important role of Zn in an array of physiological processes necessitates tight control on the intracellular Zn level [14]. Cells have evolved a sophisticated system to maintain Zn homeostasis. In mammalian cells, two Zn transporter protein families, SLC39A (ZIP, ZRT/IRT-related protein) and SLC30A (ZnT) [15], serve as regulators of Zn uptake, efflux, and intracellular compartmentalization. ZIPs may increase cellular Zn content by taking up Zn into the cytosol across the plasma membrane, while ZnTs are believed to facilitate the efflux of Zn from cells or into intracellular organelles, thus decreasing the intracellular Zn level [16]. The ZIP family contains 14 human sequences, while the ZnT family contains 10 human sequences: these sequences exhibit unique tissue-specific expression and differential responsiveness to various physiologic stimuli [17–19].

Zn transporter members have recently been implicated in several human malignancies. The cell-proliferating effects of labile Zn and Zn transporters are tissue-specific. In mammary gland tumor cells, low levels of ZnT1, which lead to a higher concentration of Zn than that in normal cells, have been detected [20]. The downregulation of ZIP1 and ZIP4 is known to be a potential mechanism for the dramatically diminished Zn concentrations present in prostate cancer [21]. Conversely, ZIP4 mRNA expression is upregulated by nearly 6 times in human pancreatic cancer; the overexpression of ZIP4 promotes pancreatic cancer growth and metastasis [22–24]. Likewise, ZIP6 and ZIP10 have been correlated with breast cancer metastasis and may play a causal role [25, 26]. ZIP7, which is located at a critical node in Zn-mediated tyrosine kinase signaling, might be a novel target for cancer treatment [27]. However, few studies have explored the expression of Zn transporters and their specific roles in lung cancer.

In light of the importance of Zn and Zn transporters in the development and progression of human cancers, we investigated whether dysregulated expression of Zn transporters contributes to lung tumorigenesis. In the present study, we examined the gene profile of 24 Zn transporters (14 ZIPs and 10 ZnTs) in lung cancer cell lines, normal human lung cells, and non-small cell lung cancer tissue specimens, to further characterize the involvement of Zn transporters and identify the potential key Zn transporters that may play important roles in human lung cancer.

## RESULTS

### Expression of Zn transporters in lung cancer cell lines

**Figure 1:**
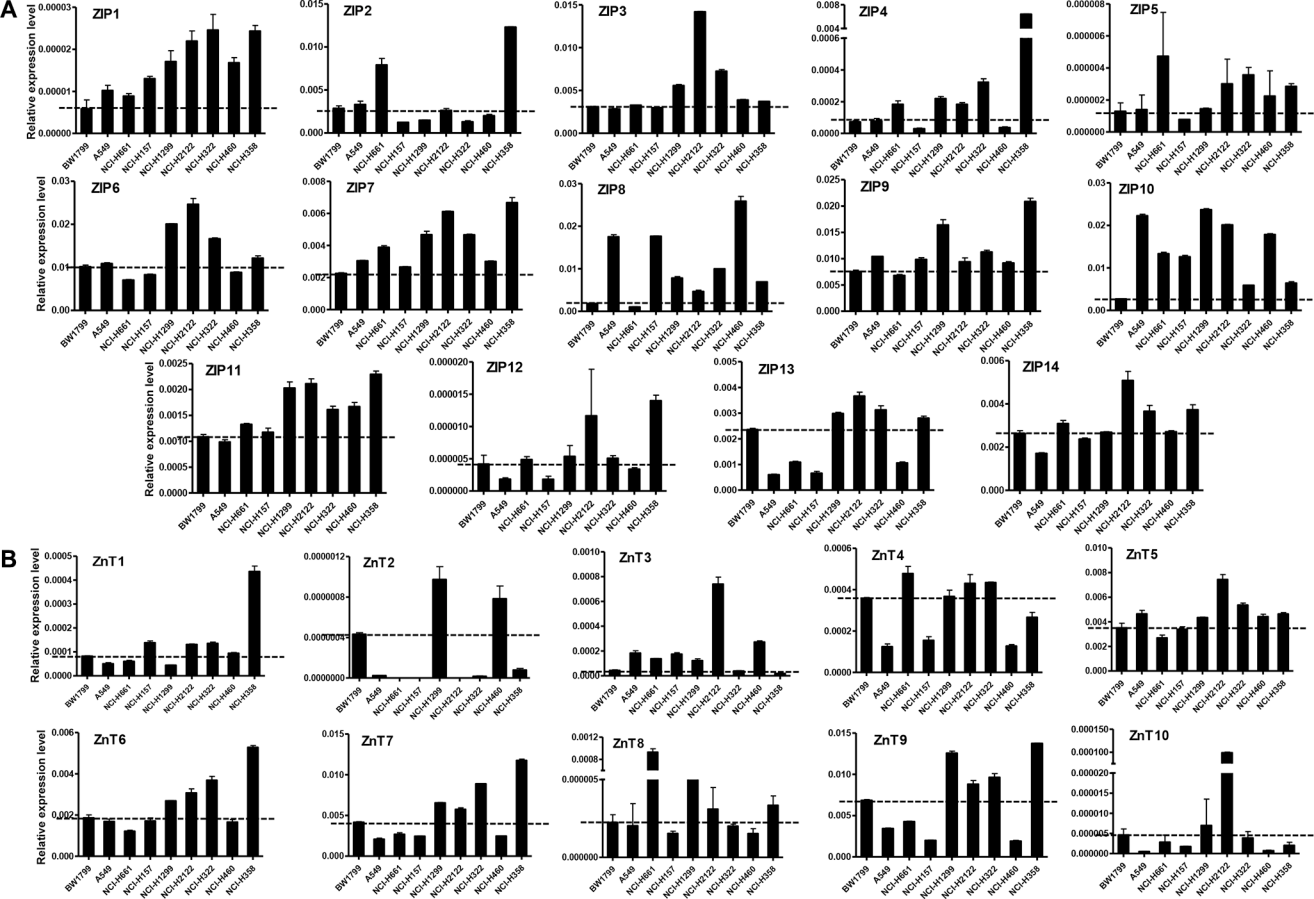
Relative mRNA expression of Zn transporters in lung cancer cell lines (**A**) ZIPs. (**B**) ZnTs. The relative mRNA expression level was shown in y-axis, which was calculated and normalized to β-actin.

We examined the expression levels of all 14 ZIPs and 10 ZnTs mRNA in eight different human lung cancer cell lines, using normal BW1799 cells as controls (Figure 1). ZIP2 was overexpressed in three cell lines (A549, NCI-H661, NCI-H358), while all other ZIPs were overexpressed in at least four cell lines (Figure 1A). Elevated levels of ZIP1, ZIP7, and ZIP10 were found in all eight lung cancer cell lines. The mRNA levels of the 14 ZIPs were all increased in NCI-H358 cells. ZIP4, which was increased by 160 folds in NCI-H358 cells, was the most highly expressed ZIP gene.

Most ZnTs, except ZnT2 and ZnT10, were upregulated in four cell lines. ZnT2 was overexpressed in NCI-H1299 and NCI-H460 cells, while ZnT10 was overexpressed in NCI-H1299 and NCI-H2122 cells (Figure 1B). Two cell lines, NCI-H1299 and NCI-H2122, had relatively high mRNA levels of most ZnTs. Previous studies demonstrated that ZnT6 is predominantly localized in the lung. Our results revealed four cell lines (NCI-H1299, NCI-H2122, NCI-H433, and NCI-H358) with ZnT6 mRNA levels increased by 50% to 150%.

### Expression profile of Zn transporters in non-small cell lung cancer tissues

**Figure 2:**
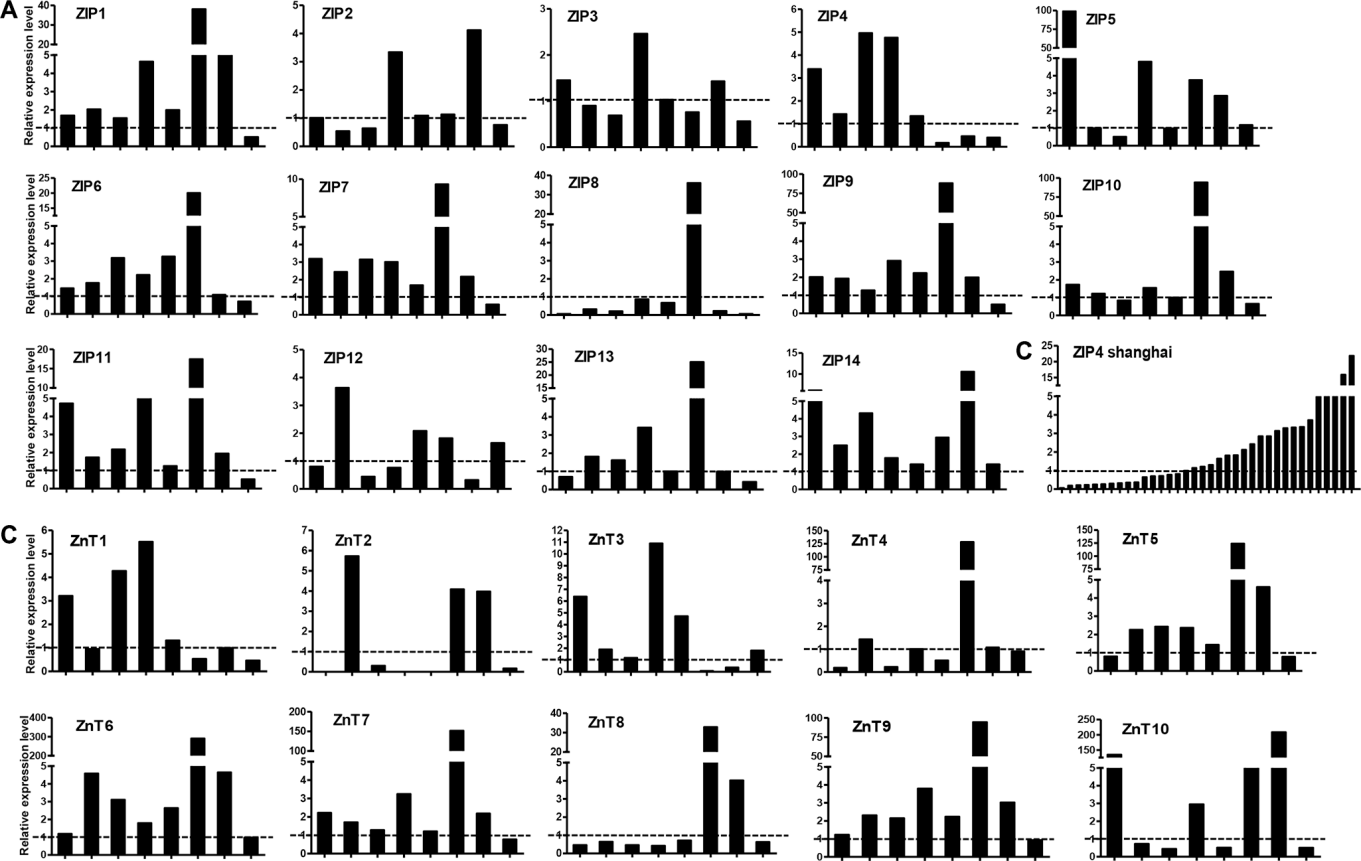
Relative mRNA expression of Zn transporters in lung cancer tissues (**A**) ZIPs in the UT Health cohort. (**B**) ZIP4 in the Shanghai cohort. (**C**) ZnTs in the UT Health cohort. Y-axis indicated fold change of gene expression (tumor versus non-tumor). X-axis indicated each lung cancer tissue samples. The relative expression was calculated and normalized to β-actin.

We observed differential overexpression levels of Zn transporters in the patients of the UT cohort (Figure 2). The mRNAs of six Zn transporters (ZIP1, ZIP6, ZIP7, ZIP9, ZIP11, and ZIP14) were overexpressed in the majority of the patients (6 or 7 out of 8 patients, Figure 2A). The mRNAs of seven Zn transporters (ZIP2, ZIP3, ZIP4, ZIP5, ZIP10, ZIP12, and ZIP13) were overexpressed in at least half of the patients (4 or 5 out of 8 patients, Figure 2A). ZIP8 was downregulated in most of the cancer patients except for one patient.

**Figure 3:**
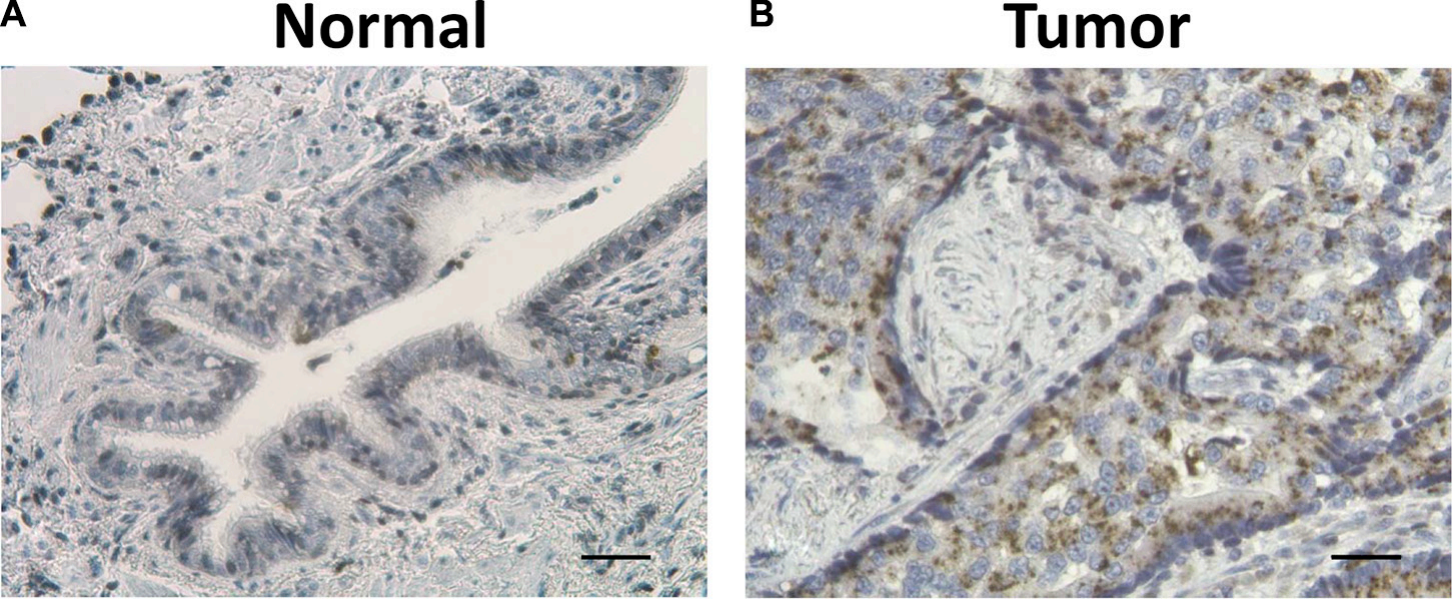
Representative hematoxylin (HE) and ZIP4 IHC staining in surrounding normal tissues (A) and lung cancer tissues (B) Tissue sections were stained using ZIP4-specific Ab. The brown color indicates positive ZIP4 staining with the cell nuclei stained blue by haemotoxylin. Scale bar represents 100 μm.

Considering the confirmed role of ZIP4 in the carcinogenesis of pancreatic, liver cancer, and glioblastoma multiform (GBM) [22, 23, 28–30], we further conducted a similar investigation with expanded samples of 36 paired specimens of tumor and surrounding benign tissues from a separate cohorts of patients who had non-small cell lung cancer. We found that 58.3% (21/36) of patients possessed higher ZIP4 mRNA levels in tumor tissues, which were normalized to the matched surrounding non-tumor tissues (Figure 2B). In contrast, 62.5% (5/8) patients in the UT cohort exhibited ZIP4 overexpression. This finding was consistent between these two cohorts (*P* = 1.00). IHC staining also indicated that ZIP4 was overexpressed in tumor tissues (Figure 3).

ZnTs expression was relatively low in most tissue samples (Figure 2C). ZnT5 and ZnT6 were overexpressed in tumor tissues from 75% (6/8) of the patients, while ZnT7 and ZnT9 were overexpressed in tumor tissues from 87.5% (7/8) of the patients. The mRNA levels of all other ZnTs in majority tumor tissues showed a downregulated expression tendency, when compared with those levels in the surrounding non-tumor tissues.

## DISCUSSION

Recent studies have indicated that Zn transporters and Zn homeostasis play important roles in malignant development and progression, especially in prostate, pancreatic, liver, and breast cancers. The relationship between Zn transporters and other cancers is a topic of great interest. Until now, little research has focused on Zn transporters in lung cancer. In the current study, we examined the gene profiles of Zn transporters in both lung cancer cell lines and human tissue samples. The results demonstrated variations in ZIPs and ZnTs expressions across cell lines and human lung cancer specimens. ZIPs were mostly upregulated, while ZnTs exhibited a downward expression trend. One of the key Zn transporter ZIP4 is actively expressed and showed upregulation in non-small cell lung cancer. Taken together with the results from our previous studies, our finding of constitutively high levels of ZIP4 mRNA and protein in lung cancer suggests that aberrant activation of the ZIP4 gene may play an important role in tumorigenesis of lung cancer, and shows promise as a potential therapeutic target.

Zn transporters possess both tissue-specificity and ubiquitous expression patterns within the human body. Such differential expression and subcellular localization suggests that Zn transporters may exhibit diverse and unique effects on cell biology and physiology. For example, ZnT1 displays a ubiquitous tissue distribution and is more highly expressed in tissues involved in Zn acquisition, recycling, or transfer, such as the small intestine [31, 32], consistent with its function as a major Zn exporter in the body. ZnT2, ZnT3, ZnT4, and ZnT8 are predominantly expressed in secretory tissues, such as mammary glands, glutamatergic neurons, the prostate, and pancreatic β-cells [33]. ZnT5, ZnT6, ZnT7, ZnT9, and most ZIPs are widespread and show relatively high expression levels in certain tissues (Table 1), consistent with our previous results [34]. ZnT10 is mainly expressed in brain, retina, and liver tissues [35]. ZIP4 and ZIP5 show a similar pattern of tissue-specific expression, with high expression in the liver, kidney, pancreas, and throughout the small intestine and colon [36]. Multiple studies and tissue array data showed that the greatest amount of ZIP14 is expressed in the liver, followed by the intestine [37, 38]. In the present study, ZIP4 was also overexpressed in six lung cancer cell lines and in 59% (26/44) of paired lung cancer and normal tissue specimens, supporting the notion that the ZIP4-induced tumor-promoting effect may participate in lung cancer carcinogenesis.

**Table 1:** Summary of the Zn transporter family members

Gene name	Alias	Tissue/cell distribution	Subcellular localization	Clinical significance
SLC30A1	ZnT1	Widespread	Plasma membrane	Associated with embryonic death [32]
SLC30A2	ZnT2	Mammary gland, prostate, retina, pancreas, small intestine, and kidney	Endosomal/ lysosomal/ secretory vesicles, plasma membrane, and mitochondria	Reduce zinc content in nursing women [31]
SLC30A3	ZnT3	Brain, testes, and pancreas	Synaptic vesicle	Seizures, learning deficits, and memory loss [18]
SLC30A4	ZnT4	Widespread, predominant in mammary gland, placenta, prostate, brain, and kidney	Endosomal/ secretory vesicles, and plasma membrane	Lethal milk [31]
SLC30A5	ZnT5	Widespread, predominant in heart, placenta, pancreas, prostate, ovary, testis, small intestine, thymus, and bone	Golgi, unknown vesicles, and plasma membrane	Bone abnormalities and heart failure [16]
SLC30A6	ZnT6	Widespread, predominant in brain, lung, and intestine	Golgi, and unknown vesicles	Alzheimer's disease [13]
SLC30A7	ZnT7	Widespread, predominant in intestine, stomach, prostate, retina, pancreas, testis, and muscle	Golgi, and unknown vesicles	Prostate cancer, low adiposity, and diet-induced diabetes [17]
SLC30A8	ZnT8	Pancreas, thyroid, adrenal gland, and testis	Secretory granule	Diabetes [19]
SLC30A9	ZnT9	Widespread	Cytoplasm, nucleus	Lower in obese women [33]
SLC30A10	ZnT10	Brain, retina, and liver	Unknown	Contribute to the progression of Alzheimer's disease [14]
SLC39A1	ZIP1/ ZIRTL	Widespread	Plasma membrane and intracellular vesicles	Prostate cancer and neurodegeneration [11]
SLC39A2	ZIP2/ Eti-1/ 6A1	Widespread	Plasma membrane	Involved in keratinocyte differentiation [18]
SLC39A3	ZIP3	Widespread, mammary cells, and testis	Plasma membrane and lysosomes	Neurodegeneration [31]
SLC39A4	ZIP4	Gastrointestinal tract, kidney, and hippocampal neurons	Plasma membrane, lysosomes, and apical surface of enterocytes	Acrodermatitis enteropathica, pancreatic cancer, and liver cancer [22, 30]
SLC39A5	ZIP5/ LZT-Hs7	Pancreas, kidney, liver, stomach, and intestine	Plasma membrane, lysosomes, and basolateral surface of enterocytes	Alcoholic liver disease [16]
SLC39A6	ZIP6/ LIV1	Widespread	Plasma membrane	Breast, pancreatic, cervical and prostate cancers, and neuroblastoma
SLC39A7	ZIP7/ HKE4	Widespread	ER, Golgi, and intracellular vesicles	Breast cancer [27]
SLC39A8	ZIP8/ BIGM103/ LZT-Hs6	Widespread, predominant in T cells, erythroid, and testis	Plasma membrane, lysosomes, and mitochondria	Inflammation, breast cancer, and cadmium-mediated toxicity [16]
SLC39A9	ZIP9	Widespread	Trans-Golgi	Prostate cancer and breast cancer
SLC39A10	ZIP10/ LZT-Hs2	Brain, liver, erythroid, and kidney	Plasma membrane	Breast cancer [26]
SLC39A11	ZIP11	Testis and digestive system	Cytoplasm and nuclei	Help maintain mucosal integrity and function [31]
SLC39A12	ZIP12	Brain, lung, testis, and retina	Unknown	Essential for neurulation and neuronal differentiation [31]
SLC39A13	ZIP13	Widespread	Intracellular vesicles and Golgi	Ehlers-Danlos syndrome (SCD-EDS) [18]
SLC39A14	ZIP14	Widespread	Plasma membrane	Asthma, inflammation, and colorectal cancer [38]

Early data revealed significantly lower Zn content and higher Cu/Zn ratio in serum from patients with lung cancer than that from patients in serum with nonmalignant lung diseases or normal healthy controls. Progressive changes were observed with advanced stages of disease and postoperative survival of lung malignancy [39–41]. Later studies demonstrated that dietary Zn intake was associated with a lower risk of lung cancer [42–45]. However, no research has focused on the significance of Zn and Zn transporters in lung tumorigenesis, even though there are considerable data indicating that Zn transporters play definite tumor-promoting roles in many cancers.

To date, ZIP4 is the only mammalian SLC39A superfamily member that has been shown to be an essential zinc transporter and is involved in the adaptation to Zn deficiency [30]. Consequently, ZIP4 may also mediate carcinogenesis. This hypothesis was confirmed in prostate cancer [46], pancreatic cancer [22–24, 34, 47, 48], and hepatocellular carcinoma [30]. This concept is also supported by the results from a meta-analysis of microarray data deposited in the Oncomine and Geo databases for ZIP4 (SLC39A4) expression. In the Oncomine database, ZIP4 was found to be among the top 10% of genes expressed in many human cancers and was associated with an advanced tumor stage. In the Geo database, ample data on SLC39A4 suggested that elevated ZIP4 mRNA was found in lymphoma, melanoma, and metastatic colon cancer. Meanwhile, the TCGA database also indicated that higher ZIP4 mRNA expression was associated with poorly differentiated lung cancer, lower probability of survival, and shorter overall survival; while lower ZIP4 mRNA expression was predictive for well-differentiated lung cancer, higher probability of survival, and longer overall survival. However, few studies have investigated the functional relevance between Zn transporters and lung cancer. Table 2 displays the information regarding ZIP4 expression in lung cancer from different databases. In the present study, our results showed that the differential expression of ZIP4 level may serve as a biomarker for lung cancer. However, the ZIP4-related molecular mechanism should be thoroughly examined.

**Table 2:** Lung cancer datasets showing increased ZIP4 expression

Dataset	Tumor type	Case	Sample type	Fold change	*P* value	Journal	Year
Su [50]	Adenocarcinoma	318	mRNA	2.448	2.15E-6	BMC Genomics	2007
Hou [51]	Adenocarcinoma	780	mRNA	2.134	4.94E-10	PLoS one	2010
Landi [52]	Adenocarcinoma	132	mRNA	1.761	2.75E-18	PLoS one	2008
Bittner	Non-small cell lung carcinoma	1142	mRNA	1.364	0.011	No	2012
TCGA	Adenocarcinoma	1325	mRNA	1.158	1.73E-14	No	2012

In summary, our study described a complete gene profiling of Zn transporters (14 ZIPs and 10 ZnTs) in human lung cancer tissues and cell lines. The expression of Zn transporters, particularly ZIP4, was dysregulated in non-small cell lung cancer. These findings may provide new insight into understanding the lung cancer pathogenesis and developing new targeted therapies. Further studies of the underlying biologic mechanisms of the aberrantly expressed ZIP4 and Zn transporters in lung cancer are warranted.

## MATERIALS AND METHODS

### Chemicals and reagents

The iQ SYBR Green supermix and iScript cDNA synthesis kits were purchased from Bio-Rad (Hercules, CA). The RNAqueous-4PCR and DNA removal kits were obtained from Ambion (Austin, TX). The avidin-biotin reaction (ABC) kit was ordered from Vector Laboratories (Burlingham, CA). Other chemicals were purchased from Sigma (St. Louis, MO).

### Cell culture and tissue collection

Human lung cancer cell lines A549, H460, H1299, H2122, H661, H157, H322, and H358 were purchased from the American Type Culture Collection (ATCC, Rockville, MD). All cells were maintained in RPMI 1640 containing 10% fetal bovine serum (FBS), 100 U/mL penicillin, 100 U/mL streptomycin, and 2 mM glutamine in a humidified atmosphere with 5% CO_2_ at 37°C. BW1799 normal human lung cells were kindly provided by Dr. Xiangwei Wu of the University of Texas MD Anderson Cancer Center and were maintained in Keratinocyte-SFM medium. Two independent cohorts of lung tumor tissues and surrounding non-tumor tissues were collected from patients pathologically diagnosed with non-small cell lung cancer who underwent surgery at the University of Texas Health Science Center at Houston (UTHealth cohort, *n* = 8) and Fudan University Shanghai Cancer Center (Shanghai cohort, *n* = 36), respectively. Informed consent was obtained from each patient. All experiments were performed in accordance with the Declaration of Helsinki and approved guidelines.

### RNA Extraction and real-time PCR (RT-PCR)

Total RNA was extracted from the cell lines and homogenized lung tissues using an Ambion “RNAqueous-4PCR” kit in accordance with the manufacturer's instructions. Briefly, cells or tissue samples were lysed with Ambion lysis solution for 20 min. The lysates were mixed with an equal volume of 64% ethanol, and were then transferred to an Ambion mini-column in a 2-ml collection tube and centrifuged at 10,000 × g for 1 min. The column was washed in turn with 700 μl of wash buffer 1,500 μl of wash buffer 2, and 500 μl of wash buffer 3. After incubation with 50 μl of prewarmed elution solution, the eluted fluid was collected using a new tube and recovered. We added another 50 μl of elution solution, followed by centrifugation at 10,000 × g for 1 min at room temperature. The extracted RNA was treated with DNase I to remove genomic DNA contamination using an Ambion DNA removing kit (Austin, Texas). Then, the extracted RNA was quantified by measuring absorbance (260 nm). The mRNA levels for ZIPs and ZnTs in lung cancer cells and tissues were analyzed via RT-PCR using the iCycler system (Bio-Rad, Hercules, CA) with the same primer sequences as described previously [30]. The RT-PCR reaction system included 100 nM of each primer, diluted cDNA templates, and iQ SYBR Green supermix, and was performed by running for 40 cycles at 95°C for 20 sec and 60°C for 1 min. RT-PCR efficiency was evaluated by serially diluting the template cDNA and collecting the melting curve data to assess RT-PCR specificity. All sample measurements were run in triplicate. The corresponding no-reverse transcriptase (RT) mRNA sample was included as a negative control. The β-actin primer was included in every plate to correct for sample-to-sample variation. The mRNA level of each sample for each gene was normalized to that of the β-actin mRNA. The relative mRNA level was presented as 2^ [(Ct/β-actin - Ct/gene of interest)].

### Immunohistochemical (IHC) staining

Clinical lung cancer specimens and matched adjacent non-tumor tissue specimens were collected and processed into 5-μm slices using a Cryostat (Meyer Instruments, Houston, TX). Tissue slices were incubated with homemade anti-hZIP4 antibody for 1 h at room temperature after blocking buffer was added for 30 min at room temperature. The human ZIP4 antibody was generated in rabbits against a KLH-conjugated 14-amino acid synthetic peptide as previously described [49]. After washing with PBS, the slices were incubated with biotinylated secondary antibody for 30 min, and then avidin-biotin-peroxidase solution (ABC) for 1 h at room temperature, followed by 0.1% DAB and 0.003% H_2_O_2_ in Tris-buffered saline for 5–10 min. Immune complexes were detected under a phase contrast microscope.

### Statistical analysis

Quantitative data are shown as means ± standard deviations. Significant differences between control and treatment groups were determined using independent Student's *t*-test. The data were analyzed using SPSS software 17.0. A *P* value of < 0.05 was considered statistically significant.
